# SMYD2 Promotes Calcium Oxalate-Induced Glycolysis in Renal Tubular Epithelial Cells via PTEN Methylation

**DOI:** 10.3390/biomedicines12102279

**Published:** 2024-10-08

**Authors:** Shengyu Pan, Tianhui Yuan, Yuqi Xia, Weimin Yu, Haoyong Li, Ting Rao, Zehua Ye, Lei Li, Xiangjun Zhou, Fan Cheng

**Affiliations:** Department of Urology, Renmin Hospital of Wuhan University, Wuhan 430060, China; 2016283020168@whu.edu.cn (S.P.); whuyth@foxmail.com (T.Y.); xiaxuqi1994@whu.edu.cn (Y.X.); weimin1@163.com (W.Y.); rm002243@whu.edu.cn (H.L.); tinart@126.com (T.R.); yezehua0704@163.com (Z.Y.); 973515222@aliyun.com (L.L.); zxj19840902@163.com (X.Z.)

**Keywords:** SMYD2, glycolysis, renal injury, renal fibrosis, nephrolithiasis

## Abstract

**Background/Objectives**: Damage to renal tubular cells (RTCs) represents a critical pathological manifestation in calcium oxalate (CaOx) stone disease, but the underlying mechanism remains elusive. Energy metabolism reprogramming is a vital influencer of RTC survival, and SMYD2 is a histone methylation transferase that has been extensively implicated in various metabolic disorders. Hence, this research aimed to identify whether SMYD2 induces the reprogramming of energy metabolism in RTCs exposed to CaOx nephrolithiasis. **Methods**: Kidney samples were obtained from patients who underwent laparoscopic nephrectomy for non-functioning kidneys caused by nephrolithiasis. The glyoxylate-induced CaOx stone mice model was established and treated with AZ505. The SMYD2-knockout HK-2 cell line was constructed. Histological changes were evaluated by HE, VK, Tunel, Masson stainings. The molecular mechanism was explored through co-immunoprecipitation and western blotting. **Results**: The results found that SMYD2 upregulation led to energy reprogramming to glycolysis in human kidney tissue samples and in mice with CaOx nephrolithiasis. We also identified the substantial involvement of glycolysis in the induction of apoptosis, inflammation, and epithelial–mesenchymal transition (EMT) in HK-2 cells caused by calcium oxalate monohydrate (COM). In vivo and in vitro results demonstrated that SMYD2 inhibition reduces glycolysis, kidney injury, and fibrosis. Mechanistically, SMYD2 was found to promote metabolic reprogramming of RTCs toward glycolysis by activating the AKT/mTOR pathway via methylated PTEN, which mediates CaOx-induced renal injury and fibrosis. **Conclusions**: Our findings reveal an epigenetic regulatory role of SMYD2 in metabolic reprogramming in CaOx nephrolithiasis and associated kidney injury, suggesting that targeting SMYD2 and glycolysis may represent a potential therapeutic strategy for CaOx-induced kidney injury and fibrosis.

## 1. Introduction

Nephrolithiasis is a common urological condition whose global prevalence has been on the rise [1,2]. Operation remains the mainstay of treatment for nephrolithiasis [3]. However, despite surgery being associated with a high stone-free rate, the 5-year recurrence rate remains as high as 60% [4]. The rising incidence and recurrence rates of nephrolithiasis place a substantial burden on healthcare systems worldwide, with the United States alone spending more than $2 billion annually on managing this condition [3]. Continued irritation due to the CaOx nephrolithiasis can result in renal tissue damage, fibrosis, and eventually complete renal failure [5,6,7]. Patients with nephrolithiasis have a high risk of developing chronic kidney disease (CKD) and progressing to end-stage renal disease, but the molecular processes that promote kidney stone recurrence, kidney injury, and fibrosis are unclear [8,9,10]. Therefore, it is imperative to identify methods to inhibit the recurrence of kidney stones and the associated kidney injury and fibrosis.

Kidney stones can comprise calcium oxalate (CaOx), magnesium ammonium phosphate, uric acid, or cystine, with CaOx stones being the most frequently encountered in clinical practice, i.e., 80% of cases [11]. The formation of nephrolithiasis follows through a series of processes, including crystal nucleation, growth, aggregation, and retention [12,13,14]. Retained CaOx crystals can lead to necrosis and apoptosis of renal tubular cells (RTCs), either directly or indirectly [15,16,17]. Damage to these cells exacerbates renal injury and fibrosis and contributes to renal stone formation [18,19]. Therefore, it has come to be identified that the fate of RTCs is a crucial determinant of renal stone recurrence.

Metabolic reprogramming has been related to the development and progression of several kidney diseases [20,21,22,23,24]. An elevated renal cortical glucose uptake in the presence of nephrolithiasis suggests potential metabolic reprogramming in the kidney tissue [7]. However, the specific metabolic changes and the role of RTCs in experimental models of CaOx nephrolithiasis are poorly understood. We hypothesize that RTCs exposed to CaOx nephrolithiasis undergo metabolic reprogramming towards glycolysis, contributing to stone formation, kidney injury, and fibrosis.

Epigenetics plays a role in the development as well as prevention of several renal diseases [25,26,27,28,29]. SMYD2, a member of the SMYD family of lysine methyltransferases with SET and MYND domains, methylates a wide range of histone and non-histone substrates, resulting in various biological effects [30]. SMYD2 promotes adipose differentiation by phosphorylating STAT3, highlighting the significance of SMYD2 in lipid metabolism [31]. Analysis using an experimental model of diabetic nephropathy showed that Ranunculaceae extracts inhibit SMYD2 expression and attenuate renal fibrosis, underscoring the strong association between SMYD2 and metabolism-related disorders [32]. Nevertheless, the function of SMYD2 in CaOx nephrolithiasis remains unclear. Thus, this study aimed to explore whether SMYD2 mediates renal injury and fibrosis by regulating metabolic reprogramming in RTCs.

The present study determined that RTCs undergo metabolic reprogramming towards glycolysis in CaOx nephrolithiasis, with glycolysis being critical for kidney injury and fibrosis. SMYD2 activation promotes glycolysis in RTCs and contributes significantly to CaOx-induced renal injury, fibrosis, and renal stone recurrence. 

## 2. Materials and Methods

### 2.1. Clinical Specimen

The Ethics Committee of Renmin Hospital of Wuhan University granted consent for this study (approval number: WDRY2021-KS047). Clinical specimens in the kidney stone group (KS, *n* = 6) were acquired from individuals who underwent laparoscopic nephrectomy for non-functioning kidneys caused by nephrolithiasis. Specimens in the control group (CON, *n* = 6) were acquired from non-tumorous renal tissues of individuals who underwent laparoscopic partial or radical nephrectomy.

### 2.2. Mouse Model

Male C57BL/6 black mice (26–28 g, 6–8-week-old) were purchased from the Hubei Provincial Centre for Disease Control and Prevention (Wuhan, China). Following published protocols [5,33], a mouse model of glyoxylate-induced CaOx stones was established. Fifteen mice were divided into three groups: the control group (CON), the glyoxylate group (Gly), and the combined glyoxylate and AZ505 group (Gly+AZ505). Mice in the CON group received daily intraperitoneal injections of saline, those in the Gly group received daily injections of glyoxylate (100 mg/kg/day) for 12 consecutive days, and those in the Gly+AZ505 group received daily injections of glyoxylate (100 mg/kg/day) and AZ505 (10 mg/kg, HY-15226, MedChemExpress, Monmouth Junction, NJ, USA) for 12 consecutive days. The dose of AZ505 was selected based on the literature [29,34]. After the termination of the experiment, kidney tissues and blood were collected before the euthanasia of the mice. All animal experiments were conducted following the approval granted by the Animal Care and Ethics Committee of Renmin Hospital of Wuhan University (Approval No. WDRM-20200604).

### 2.3. Cell Culture and Transient Transfection

Human renal tubular epithelial cells (HK-2) were cultured in culture plates with DMEM (Gibco, Waltham, MA, USA) supplemented with 10% fetal bovine serum (FBS, Gbico) in an incubator at 37 °C with 5% CO_2_. After reaching 80% cell density, calcium oxalate monohydrate (COM) (100 μg/mL) was given, and the cells were incubated for 12, 24, and 48 h to establish the model and conduct the assays. SMYD2 silencing was achieved by transfection HK-2 cells with SMYD2 siRNA (GenePharma, Shanghai, China) via Lipofectamine 3000 (Invitrogen, Waltham, MA, USA) according to the manufacturer’s protocol. The sense strand of the SMYD2-siRNA sequence is 5′- GGUGACGCGUCUCCAAUAACATT -3′, and the antisense strand is 5′- UGUUUUAUUGGAGACGCGUCACCTT -3′.

### 2.4. Histopathological Examination in the Kidney

Kidney samples were fixed in 4% paraformaldehyde, embedded in paraffin, and sectioned at 4 µm. The sections were rehydrated and subjected to hematoxylin–eosin (HE) staining, Masson’s trichrome staining, Von Kossa (VK) staining, and Oil Red O staining to evaluate renal injury, CaOx crystal deposition, fibrosis, and lipid accumulation, respectively. Renal injury was assessed using the renal tubular injury score as described previously [5]. Subsequently, staining indices were quantified using ImageJ software, version 1.52i (National Institutes of Health, Bethesda, MD, USA).

### 2.5. Immunofluorescence

Sections of formalin-fixed, paraffin-embedded human and mouse kidney samples or HK-2 cells were stained with primary antibodies against SMYD2 (1:500, 21290-1-AP, Proteintech, Wuhan, China), HK2 (1:200, A0994, ABclonal, Wuhan, China), PKM2 (1:200, A18799, ABclonal, Wuhan, China), LDHA (1:200, A1146, ABclonal), CD68(1:100, ba3638, Boster, Wuhan, China), and F4/80 (1:500,ab300421, Abcam, Cambridge, UK). The sections were then incubated for 1 h at 37 °C with fluorescence-labeled secondary antibodies (Proteintech, Wuhan, China). Lastly, the slides were examined using fluorescence microscopy (Nikon, Tokyo, Japan). The relative expression levels were analyzed using ImageJ software.

### 2.6. Immunohistochemistry

Kidney specimens from humans and mice were sectioned and subjected to formalin fixation and paraffin embedding. The sections were then incubated overnight at 4 °C with antibodies against HK2 (1:200, A0994, ABclonal), PKM2 (1:500, 60268-1-Ig, Proteintech), LHDA (1:200, A21741, ABclonal), PPARα (1:100, 66826-1-Ig, Proteintech), CPT1 (1:500, 15184-1-AP, Proteintech), FN(1:200,MA1116, Boster) and α-SMA (1:2000, 14395-1-ap, Proteintech). Subsequently, the samples were evaluated using an HRP-conjugated polymer system (K5007, Dako, Germany) and a Leica DM2000 scanner (Leica Biosystems, Wetzlar, Germany). The relative expression levels were analyzed using ImageJ software.

### 2.7. Western Blotting

Kidney tissues and HK-2 cells were lysed using radioimmunoprecipitation assay lysis buffer supplemented with phenylmethylsulfonyl fluoride and a phosphatase inhibitor (Servicebio, Wuhan, China). The lysed proteins underwent SDS-PAGE electrophoresis and were transferred to polyvinylidene fluoride (PVDF) membranes. The membranes were then blocked for 2 h at 25 °C. Subsequently, the membranes were incubated overnight at 4 °C with primary antibodies against SMYD2 (Proteintech), HK2 (ABclonal), PKM2 (Proteintech), LDHA (ABclonal), FN (Boster), α-SMA (Proteintech), COL1A1 (Affinity Biologicals, Ancaster, Canada), BAX (ABclonal), BCL-2 (ABclonal), Cleaved caspase3 (Abcam), mTOR (Proteintech), p-mTOR (Proteintech), S6 (ABclonal), p-S6 (ABclonal), AKT (Proteintech), p-AKT (Proteintech), PTEN (ABclonal), N-cadherin (ABclonal), Vimentin (Proteintech), Vinculin (ABclonal), and β-Actin (Servicebio). After washing with TBST, the membranes were incubated with the corresponding secondary antibody for 1 h at 25 °C. The target bands were visualized using a ChemiDoc MP imaging system (Bio-Rad, USA) and an ECL kit (Bioshap, Hefei, China). Protein expression levels were semi-quantitatively analyzed using ImageJ software.

### 2.8. Quantitative Real-Time PCR (qPCR)

The qPCR was performed following the protocols described in our previously published study [5]. All primers were as follows: β-actin, forward primer 5′-GTGCTATGTTGCTCTAGACTTC-3′, reverse primer 5′-ATGCCACAGGATTCCATACC-3′; TNF-α, forward primer 5′-GTAGCCCACGTCGTAGCAAA-3′, reverse primer 5′- ACAAGGTACAACCCATCGGC-3′; IL-1β, forward primer 5′-GCCACCTTTTGACAGTGATG-3′, reverse primer 5′-GATGTGCTGCTGCGAGATTT-3′; IL-6, forward primer 5′-GAGACTTCCATCCAGTTGCCT-3′, reverse primer 5′-TGGGAGTGGTATCCTCTGTGA-3′.

### 2.9. Enzyme-Linked Immunosorbent Assay (ELISA)

The TNF-α(EK182HS-AW1, MultiSciences, Hangzhou, China), IL-6(EK1153-AW1, MultiSciences), and IL-1β (EK101B-AW1, MultiSciences)kit are utilized for determining the levels of inflammatory factors in cell supernatant.

### 2.10. TUNEL Staining

Apoptosis was assessed using an apoptosis detection kit (C1086, Beyotime, Shanghai, China). Paraffin sections were initially dewaxed and hydrated, followed by the addition of a membrane-breaking working solution. After equilibration at room temperature, the TUNEL mix was introduced, and the specimens were incubated for 1 h at 37 °C in a thermostat. Finally, DAPI was introduced, and the specimens were incubated for 10 min in a light-proof room at room temperature. Five randomly selected magnified areas (200×) were used for each section, and positive staining was quantified.

### 2.11. Measurement of Lactate Dehydrogenase (LDH)

The concentration of lactate released from the culture broth was quantified using the Lactate Assay Kit (A019-2-1; Nanjing Jiancheng Bioengineering Institute, Nanjing, Chian). The LDH content was determined spectrophotometrically at 530 nm. Lactate concentration in the cell supernatant was measured using the instructions provided by the manufacturer.

### 2.12. Co-Immunoprecipitation

HK-2 cells were harvested, and an immunoprecipitation (IP) lysate (L-7101, Biolinkedin, Shanghai, China) was added. Anti-SMYD2 and anti-PTEN antibodies were conjugated to Protein A/G beads (L-1204, Biolinkedin) and incubated overnight on a shaker at 4 °C. The next day, the Protein A/G beads were collected and washed thrice with IP buffer to remove immune complexes from the beads. Subsequently, the immune complexes were boiled for 5 min with loading buffer, followed by Western blot detection.

### 2.13. Flow Cytometry for Detection of Apoptosis

Apoptosis was detected using the Apoptosis Detection Kit (556570; BD Biosciences, San Jose, CA, USA). Briefly, 5 μL annexin V-FITC was added to the cell suspension, and samples were well-mixed and incubated for 10 min in dim light, followed by adding 5 μL PI, thorough mixing and incubation for 5 min in dim light. The results were analyzed using a FACScan flow cytometer (BD Biosciences).

### 2.14. Cell Viability Assay

After HK-2 cells were processed, Cell Counting Kit-8 (CCK-8, Beyotime Biotechnology, Shanghai, China) was added and incubated at 37 °C for 1 h. The absorbance of each well was measured at 450 nm using an enzyme marker.

### 2.15. Statistical Analysis

All data are presented as mean ± standard deviation. Statistical analyses were conducted using Prism 9.0 GraphPad software (San Diego, CA, USA). To determine differences between the two groups, t-tests were used, and one-way analysis of variance (ANOVA) was used to test for differences among samples in multiple groups. A *p* < 0.05 was considered statistically significant.

## 3. Results

### 3.1. SMYD2 and Glycolysis Are Activated in Human Nephrolithiasis Samples

**Figure 1:**
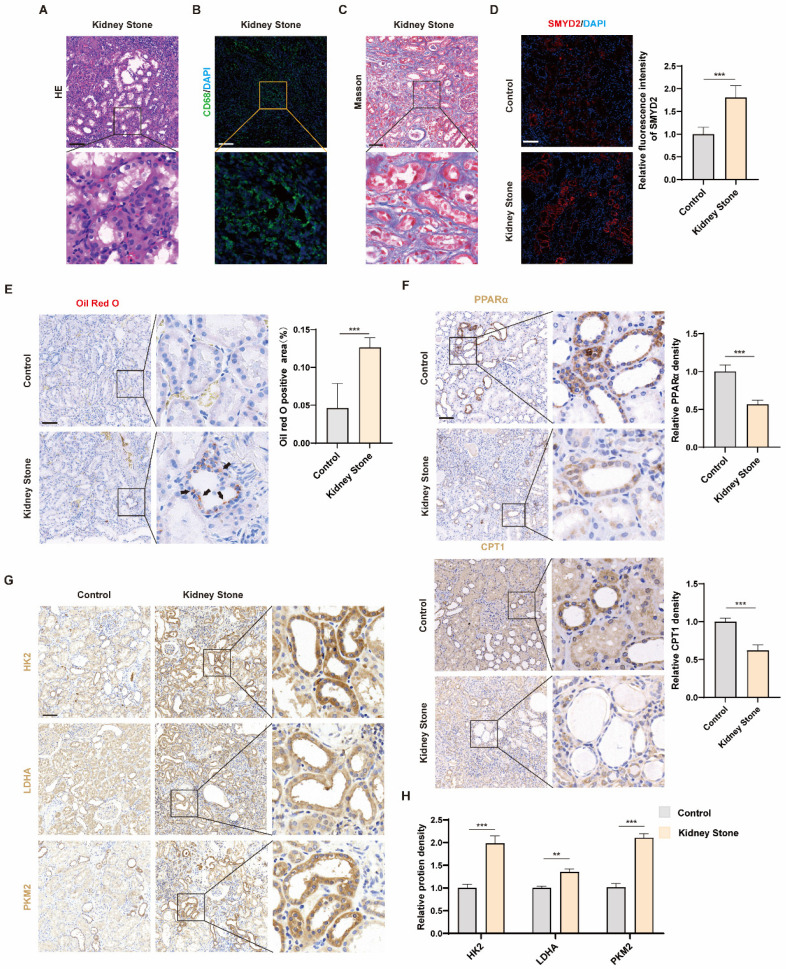
SMYD2 and glycolysis are activated in human nephrolithiasis samples. Representative images of (**A**) HE staining, (**B**) immunofluorescence for CD68, and (**C**) Masson’s staining in the KS group. (**D**) Representative immunofluorescence images and semi-quantitative analysis of SMYD2 expression. (**E**) Renal lipid metabolism was identified using Oil Red O staining, and the area of positive staining on renal sections was quantified. The black arrows point to the presence of lipid deposits in damaged RTCs. (**F**–**H**) Representative immunohistochemistry images and semi-quantitative analysis of PPARα, CPT1, HK2, LDHA, and PKM2. Scale bar = 100 μm. Data are expressed as mean ± SD, ** *p* < 0.01; *** *p* < 0.001.

We analyzed human kidney specimens to investigate the relationship between SMYD2 and nephrolithiasis and the metabolism of kidney tissues with nephrolithiasis. HE staining indicated structural disorganization in the kidneys of patients with nephrolithiasis, characterized by tubular dilatation and vacuolated epithelial cells of the renal tubules (Figure 1A). Immunofluorescence for CD68 also showed a sizeable inflammatory cell infiltrate in the kidney with nephrolithiasis (Figure 1B). Masson’s staining showed significant collagen fiber deposition in the renal interstitium (Figure 1C). Immunofluorescence analysis showed increased SMYD2 expression in human kidney specimens with nephrolithiasis compared to the control (CON) group, i.e., without nephrolithiasis (Figure 1D). SMYD2 was predominantly expressed in the cytoplasm of damaged RTCs. To assess the metabolic characteristics of the human kidney tissues with nephrolithiasis, we analyzed lipid accumulation in the damaged RTCs of the experimental group using Oil Red O staining (Figure 1E). The presence of lipid accumulation suggests a possible impairment of fatty acid oxidation (FAO) in RTCs. As expected, there was a substantial reduction in the levels of key enzymes associated with FAO (PPARα and CPT1) in the experimental group as opposed to the CON group (Figure 1F). The levels of glycolysis-associated enzymes (HK2, LDHA, and PKM2) were increased in the experimental group and localized predominantly in the cytoplasm of damaged, dilated RTCs (Figure 1G,H). Collectively, these results suggest that SMYD2 expression and glycolysis are upregulated in the cytoplasm of damaged RTCs in human kidney tissues with nephrolithiasis, indicating their association with renal injury and fibrosis.

### 3.2. SMYD2 and Glycolysis Are Activated in Glyoxylate-Induced Nephrolithiasis Mice

**Figure 2:**
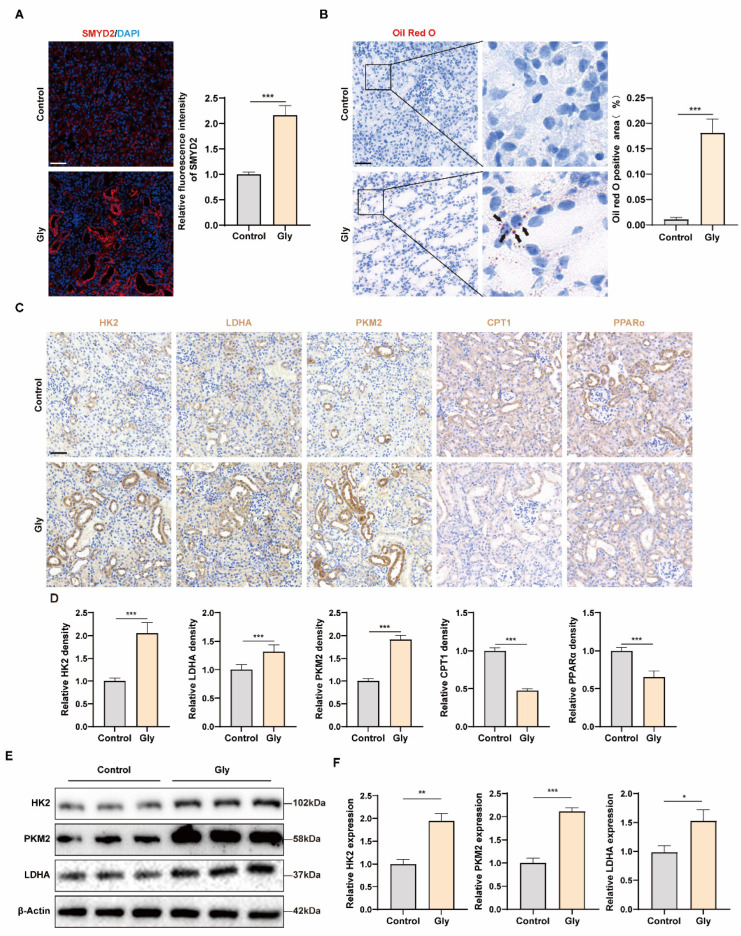
SMYD2 and glycolysis are activated in glyoxylate-induced nephrolithiasis mice. (**A**) Representative immunofluorescence images and semi-quantitative analysis of SMYD2 expression. (**B**) Representative images of Oil Red O staining and quantification of the positive staining area in renal tissues of CON and Gly groups; black arrows indicate lipid deposition in RTCs. (**C**,**D**) Representative images and semi-quantitative immunohistochemistry analysis of HK2, LDHA, PKM2, CPT1, and PPARα in CON and Gly groups. (**E**,**F**) Western blot examines HK2, PKM2, and LDHA levels in renal tissues, with quantification by densitometry. Scale bar = 50 μm. Data are expressed as mean ± SD, * *p* < 0.05; ** *p* < 0.01; *** *p* < 0.001.

To verify the results observed in human kidney specimens, we established a mouse model of CaOx crystal using glyoxylate (Gly). First, we evaluated the expression of SMYD2. Immunofluorescence analysis revealed a significant elevation in SMYD2 expression in the damaged RTCs of the Gly group compared to the CON group (Figure 2A). In the experiment to assess the metabolism of RTCs in the mouse model, we observed more severe lipid accumulation in the RTCs of the Gly group (Figure 2B), indicating impaired lipid metabolism. Immunohistochemical analysis showed increased expression of glycolysis-related enzymes and decreased expression of FAO-related enzymes in the damaged RTCs, indicating metabolic reprogramming towards glycolysis (Figure 2C,D). Furthermore, Western blot analysis demonstrated a substantial rise in the levels of HK2, PKM2, and LDHA proteins in the Gly group compared to the CON group (Figure 2E,F). These observations highlight the strong link between SMYD2 and glycolysis in the mouse model of CaOx nephrolithiasis.

### 3.3. Inhibition of SMYD2 with AZ505 Reduces Glycolysis in Glyoxylate-Induced Nephrolithiasis Mice

**Figure 3:**
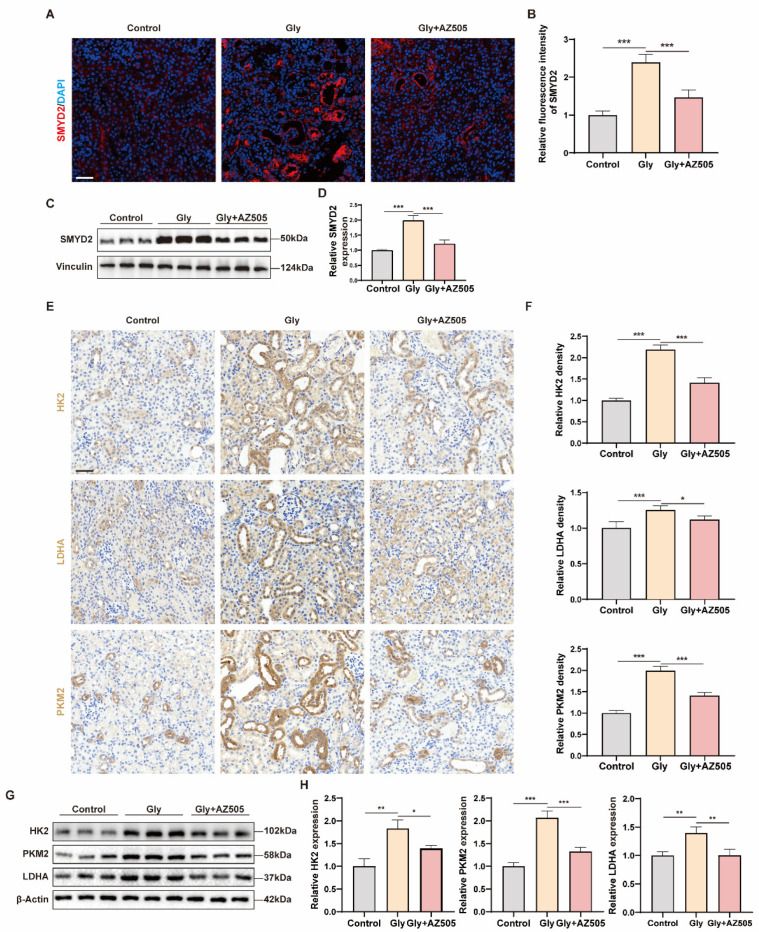
Inhibition of SMYD2 with AZ505 reduces glycolysis in glyoxylate-induced nephrolithiasis mice. (**A**–**D**) Immunofluorescence and Western blot analyses to quantify the expression of SMYD2 in renal tissues of the indicated groups. (**E**,**F**) Immunohistochemistry and semi-quantitative analysis of the levels of HK2, LDHA, and PKM2 in renal tissues of the indicated groups. (**G**,**H**) Western blot analysis and densitometric analysis of the HK2, PKM2, and LDHA levels in renal tissues. Scale bar = 50 μm. Data are expressed as mean ± SD, * *p* < 0.05; ** *p* < 0.01; *** *p* < 0.001.

Previous experimental results have suggested a close link between SMYD2 and glycolysis. To study the regulatory role of SMYD2 in glycolysis, we treated the glyoxylate-induced nephrolithiasis mice with a highly selective SMYD2 inhibitor, AZ505. AZ505 treatment effectively attenuated the glyoxylate-induced upregulation of SMYD2 expression (Figure 3A–D). Immunohistochemistry and Western blot experiments were performed to assess whether AZ505 affected the levels of glycolysis-associated enzymes. Consistent with our hypothesis, immunohistochemistry results demonstrated that AZ505 inhibited the levels of glycolysis-associated enzymes (Figure 3E,F). Western blot results also corroborated these findings (Figure 3G,H). Collectively, the results suggest that SMYD2 promotes glycolysis in vivo.

### 3.4. AZ505 Reduces CaOx Crystal Formation, Kidney Damage, Inflammation, and Fibrosis in Glyoxylate-Induced Nephrolithiasis Mice

**Figure 4:**
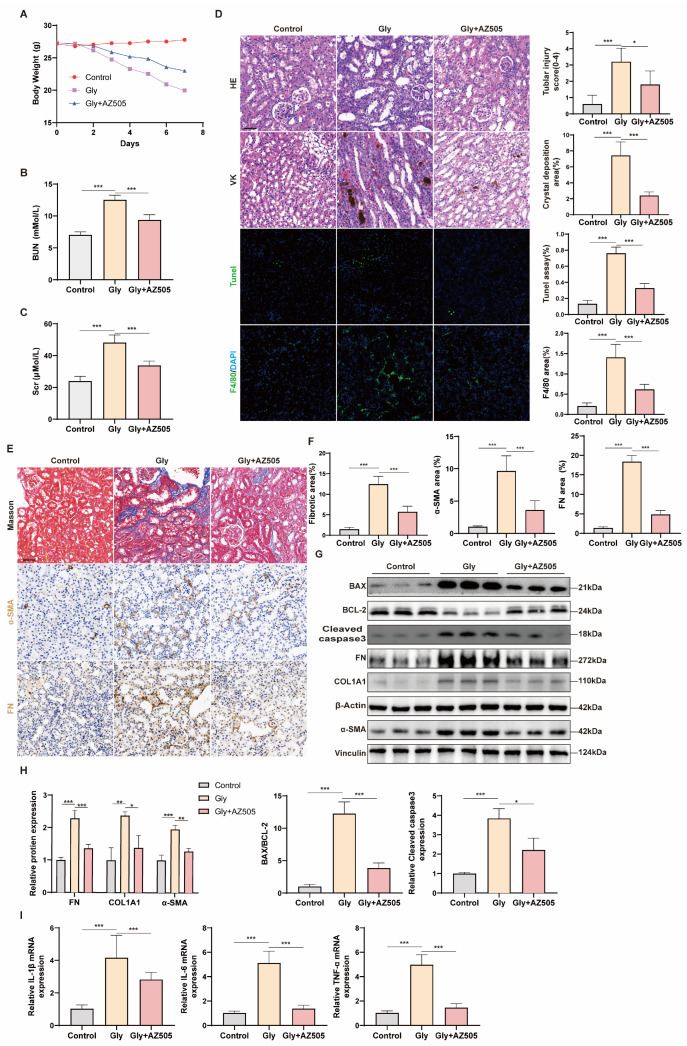
AZ505 reduces CaOx crystal formation, kidney damage, inflammation, and fibrosis in glyoxylate-induced nephrolithiasis mice. (**A**) Effect of AZ505 on body weight changes in mice. (**B**,**C**) Effects of AZ505 on serum BUN and Cr levels in mice. (**D**) Renal tubular injury, CaOx deposition, apoptosis, and inflammation were assessed by HE, VK, TUNEL staining, and immunofluorescence for F4/80, respectively, and semi-quantitative analysis. (**E**,**F**) Representative Masson’s staining, immunohistochemical (α-SMA, FN) images in renal tissues, and semi-quantitative analysis. (**G**,**H**) Western blot analysis of BCL-2, BAX, Cleaved caspase3, COL1A1, FN, and α-SMA expression in renal tissues and densitometric analysis. (**I**) The mRNA levels of IL-1β, IL-6, and TNF-α in renal tissues. Scale bar = 50 μm. Data are expressed as mean ± SD, * *p* < 0.05; ** *p* < 0.01; *** *p* < 0.001.

To examine the contribution of SMYD2 in CaOx crystal formation, kidney injury, and fibrosis, we evaluated the effects of AZ505 in a mouse model of glyoxylate-induced CaOx crystal. The glyoxylate-treated mice exhibited weight loss and raised levels of serum creatinine (Scr) and blood urea nitrogen (BUN). However, AZ505 prevented weight loss and dramatically alleviated the rise in Scr and BUN levels (Figure 4A–C). To further elucidate the effects of AZ505 on renal histology, it was observed that AZ505 mitigated renal injury, crystal deposition, apoptosis, and inflammation by using HE, VK, TUNEL staining, and immunofluorescence with F4/80 (Figure 4D). Additionally, Masson’s staining and immunohistochemistry showed that renal fibrosis was attenuated by AZ505 (Figure 4E,F). To validate these findings, we analyzed the expression of apoptosis-related proteins and fibrosis-related proteins by western blotting. AZ505 significantly inhibited the expression of Cleaved caspase3 and BAX while upregulating BCL-2 expression, thereby attenuating apoptosis (Figure 4G,H). Moreover, AZ505 inhibited the expression of α-SMA, COL1A1, and FN, thereby suppressing renal fibrosis (Figure 4G,H). Finally, AZ505 effectively inhibited the mRNA levels of IL-1β, IL-6, and TNF-α (Figure 4I).

### 3.5. Glycolysis Promotes COM-Induced Apoptosis, Inflammation, and Epithelial–Mesenchymal Transition in HK-2 Cells

**Figure 5:**
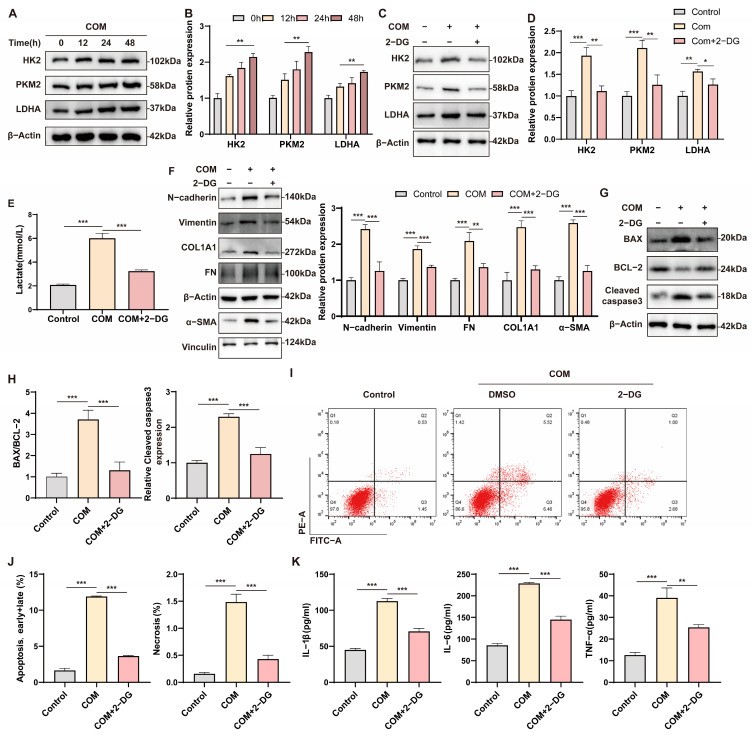
Glycolysis promotes COM-induced apoptosis, inflammation, and EMT in HK-2 cells. (**A**) HK-2 cells were stimulated with 100 μg/mL COM for 0, 12, 24, and 48 h, and the levels of HK2, PKM2, and LDHA were measured by Western blotting. (**B**) The expression of HK2, PKM2, and LDHA was quantified by densitometry. (**C**,**D**) Western blot analysis showing the levels of HK2, PKM2, and LDHA after 2-DG treatment and densitometric analysis. (**E**) Determination of lactate concentration in the supernatant of HK-2 cells. (**F**–**H**) Western blot analysis and densitometric analysis of the levels of N-cadherin, Vimentin, FN, COL1A1, α-SMA, BAX, BCL-2, and Cleaved caspase3 expression after 2-DG treatment. (**I**,**J**) Flow cytometry indicated that 2-DG inhibited apoptosis and necrosis. (**K**) The levels of IL-1β, IL-6, and TNF-α in the supernatant of the HK-2 cells in the respective groups were measured by ELISA. Data are expressed as mean ± SD, * *p* < 0.05; ** *p* < 0.01; *** *p* < 0.001.

To explore the effects of COM on RTC metabolism, we analyzed the levels of glycolysis-associated proteins in HK2 cells exposed to 100 μg/mL of COM for 12–48 h. Our results indicated that the expression of HK2, PKM2, and LDHA gradually increased under COM stimulation, peaking at 48 h (Figure 5A,B). This suggests that COM stimulation leads to a metabolic shift toward glycolysis in RTCs. Consequently, we selected 100 μg/mL of COM and an exposure duration of 48 h for subsequent experiments. The CCK8 assay demonstrated that 2-deoxy-D-glucose (2-DG) effectively rescued the decrease in cell viability induced by COM, with the most obvious improvement in cell viability observed at a concentration of 10 mM (Figure S2). To assess the effect of glycolysis inhibition on RTCs exposed to COM, we treated COM-stimulated HK-2 cells with2-DG. The results revealed that 2-DG inhibited COM-induced glycolysis and reduced lactate release (Figure 5C–E). Furthermore, 2-DG inhibited the epithelial–mesenchymal transition (EMT), preserved the RTC phenotype, and ameliorated the expression of fibrosis-associated proteins (Figure 5F). To investigate the impact of 2-DG on apoptosis, we conducted Western blotting and flow cytometric analyses. The results showed that 2-DG suppressed the upregulation of BAX and Cleaved caspase3 while increasing the expression of BCL-2 (Figure 5G,H). Additionally, flow cytometry demonstrated that 2-DG inhibited early and late apoptosis, as well as necrosis induced by COM (Figure 5I,J). Finally, we observed that 2-DG inhibited the upregulation of COM-induced IL-1β, IL-6, and TNF-α (Figure 5K). These findings suggest glycolysis is necessary to promote COM-induced apoptosis, inflammation, and EMT.

### 3.6. Silencing SMYD2 Specifically Inhibits Glycolysis, Apoptosis, Inflammation, and EMT in HK-2 Cells

**Figure 6:**
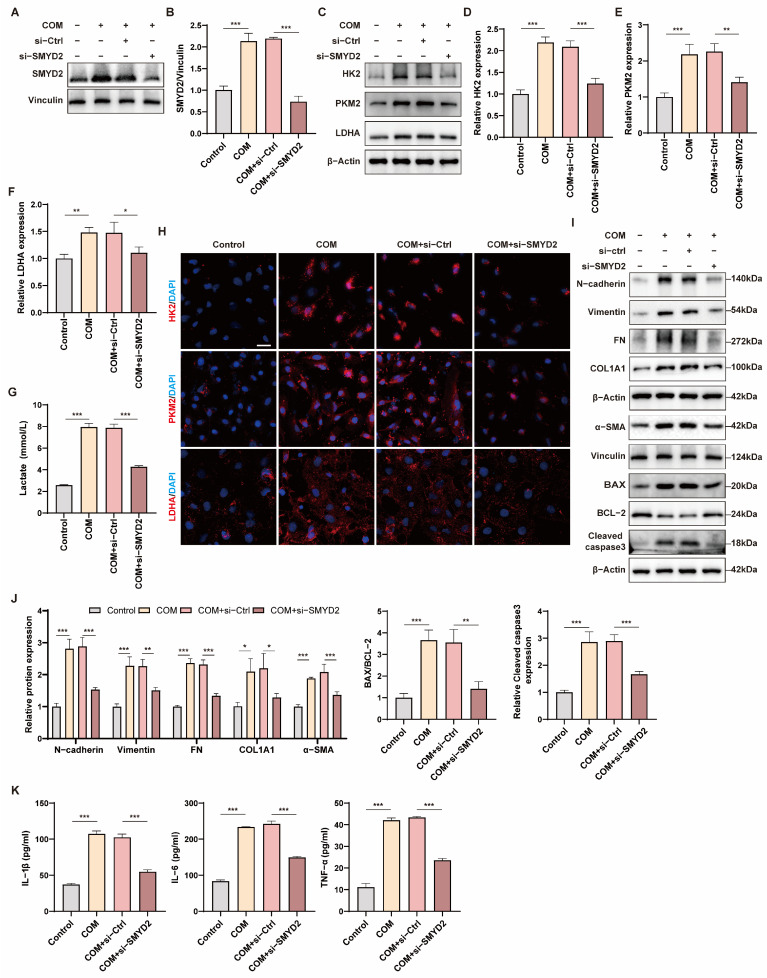
Silencing SMYD2 specifically inhibits glycolysis, apoptosis, inflammation, and EMT in HK-2 cells. (**A**,**B**) Western blot verified the knockdown efficiency of SMYD2 and quantified it by densitometry. (**C**–**F**) Western blot showed that SMYD2-knockdown suppressed HK2, PKM2, and LDHA expression. (**G**) Measurement of lactate in the supernatant of HK-2 cells from the specified groups (**H**) Representative immunofluorescence images of HK2, PKM2, LDHA after SMYD2-knockdown. (**I**,**J**) Western blot detection of N-cadherin, Vimentin, FN, Collagen l, α-SMA, BAX, BCL-2, Cleaved caspase3 expression after SMYD2 knockdown and quantification by densitometry. (**K**) The levels of IL-1β, IL-6, and TNF-α in the supernatant of HK-2 cells in the indicated group were measured by ELISA. Scale bar = 50 μm. Data are expressed as mean ± SD, * *p* < 0.05; ** *p* < 0.01; *** *p* < 0.001.

To identify the regulatory function of SMYD2 in RTCs exposed to COM, we used siRNA to silence the SMYD2 gene and examined its effects on the expression of glycolysis, apoptosis, EMT, and fibrosis-related proteins in RTCs. The knockdown efficiency of SMYD2 was assessed by Western blotting, which showed that si-SMYD2 effectively inhibited the upregulation of SMYD2 induced by COM (Figure 6A,B). The protein levels of HK2, PKM2, and LDHA were analyzed using Western blot analysis and immunofluorescence. Si-SMYD2 significantly inhibited the upregulation of HK2, PKM2, and LDHA (Figure 6C–F,H) and reduced the production of lactate in HK-2 cells after COM treatment (Figure 6G). Additionally, si-SMYD2 inhibited apoptosis and EMT, preserved the RTC phenotype, and depressed the levels of fibrosis-associated proteins (Figure 6I,J). Finally, si-SMYD2 repressed IL-1β, IL-6, and TNF-α levels and attenuated inflammation in RTCs (Figure 6K). These findings supported the hypothesis that SMYD2 regulates RTC injury and EMT through glycolysis in vitro.

### 3.7. SMYD2 Methylates PTEN and Inhibits Its Expression, Thereby Promoting Glycolysis by Activating the mTORC1 Pathway in HK-2 Cells

**Figure 7:**
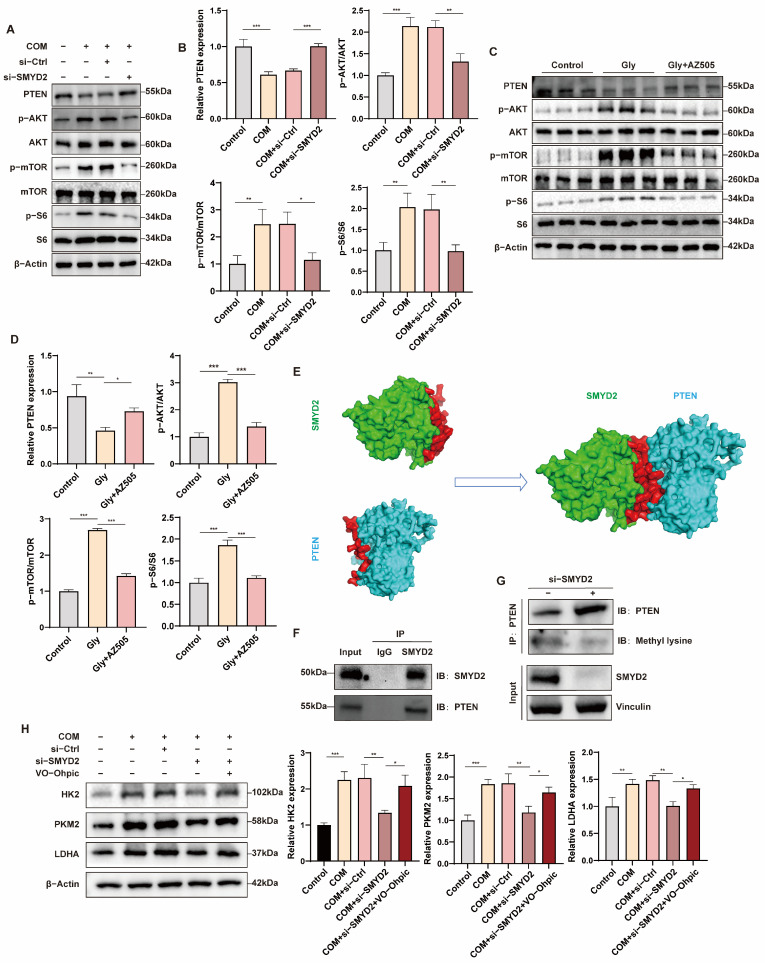
SMYD2 methylates PTEN and inhibits its expression, thereby promoting glycolysis by activating the mTORC1 pathway in HK-2 cells. (**A**–**D**) Western blot and densitometry analysis detected the expression of PTEN and mTORC1 signals after si-SMYD2 in vitro and in vivo. (**E**) Demonstration of two protein binding models in Surface form. (**F**) SMYD2 interaction with PTEN in HK-2 cells. (**G**) In HK-2 cells transfected with si-SMYD2 or si-Ctrl, IP with anti-PTEN antibody, and subsequent blotting with anti-PTEN, anti-methylated lysine antibodies. (**H**) Western blot analysis of the levels of HK2, PKM2, and LDHA in HK-2 cells. Data are expressed as mean ± SD, * *p* < 0.05; ** *p* < 0.01; *** *p* < 0.001.

The AKT/mTOR pathway is critical in regulating glucose metabolism. PTEN, a key negative regulator of AKT/mTOR, acts as a regulatory factor in glycolysis [35,36]. Thus, we hypothesized that SMYD2 activates AKT/mTOR by methylating PTEN. To verify this, we assessed PTEN expression and AKT/mTOR pathway activity via Western blotting. The results demonstrated downregulation of PTEN expression in the COM group, along with upregulation of p-AKT/AKT, p-mTOR/mTOR, and p-S6/S6, indicating AKT/mTOR pathway activation. Si-SMYD2 restored PTEN expression and inhibited the AKT/mTOR pathway in vitro (Figure 7A,B), supported by the findings from in vivo experiments (Figure 7C,D). These results suggest that SMYD2 activates AKT/mTOR signaling by regulating PTEN expression. To explore the SMYD2 regulation mechanism of PTEN, we conducted molecular docking simulations. The proteins were screened using the UniProt database, protein docking was performed using the HDOCK server, and confidence scores were calculated [37,38]. The results indicate that SMYD2 has a high probability of binding to the PTEN protein, and the binding of these two proteins was demonstrated by Surface shape (Figure 7E). Subsequent co-immunoprecipitation (CO-IP) experiments using anti-SMYD2 antibody for immunoprecipitation (IP) and anti-PTEN antibody for western blot (Figure 7F). Whether the SMYD2-PTEN interaction affects PTEN methylation was further analyzed via IP with anti-PTEN antibody and blotting with anti-methylated lysine antibody. Results showed reduced PTEN methylation in SMYD2 knockdown cells (Figure 7G), indicating the role of SMYD2 in PTEN methylation.

**Figure 8:**
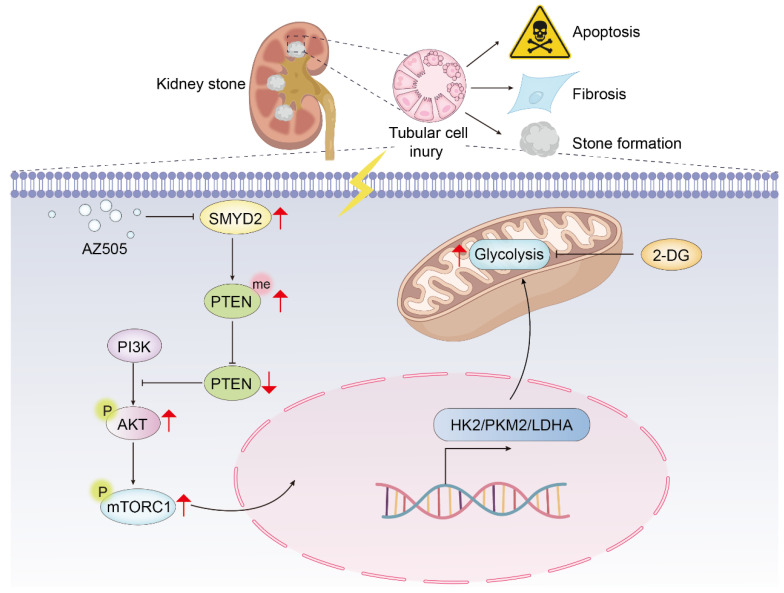
In CaOx-induced kidney injury, PTEN is methylated by SMYD2, leading to its inhibition and subsequent activation of the mTORC1 pathway. This inhibition prompts a metabolic shift toward glycolysis in RTCs, ultimately culminating in renal stone formation, kidney injury, and kidney fibrosis. Therefore, targeting the SMYD2-mediated epigenetic dysregulation of metabolism may offer a promising approach to treating renal stone disease. Inhibitors of SMYD2 and glycolysis could inhibit renal stone formation and decrease renal injury and fibrosis.

To investigate whether SMYD2 regulates glycolysis through PTEN, rescue experiments were conducted. The findings revealed that inhibition of PTEN partially rescued the loss of HK2, PKM2, and LDHA caused by si-SMYD2 (Figure 7H). In summary, the above findings indicate that SMYD2 promotes renal injury and fibrosis by enhancing glycolysis through PTEN methylation (Figure 8).

## 4. Discussion

RTC injury, inflammatory cell infiltration, and renal interstitial fibrosis are pathological changes in kidney stone disease, and current non-surgical treatments for these conditions are limited in efficacy. Renal stone formation is a complicated process, with the adhesion of crystals to cells being a central event in their formation [12,39,40,41]. However, damage to RTCs is the primary cause of crystal cell adhesion and a significant hurdle to treating this disease [15,42,43,44,45]. Consequently, understanding the molecular mechanisms by which CaOx induces damage to RTCs is fundamental to pursuing novel therapeutic targets.

The Warburg effect, characterized by the predominance of glycolysis in cancer cells even in the presence of oxygen, is closely linked to tumor cell proliferation, invasion, and metastasis [46]. Normal RTCs primarily rely on FAO for energy, with glycolysis occurring infrequently [47]. However, during transient or sustained kidney injury, there is a metabolic shift in RTCs from FAO to glycolysis. This shift is critical for maintaining the cellular energy balance, influencing signaling pathways, and modulating cellular phenotypes. Glycolysis provides energy and participates in various physiological processes, including cell proliferation, activation, oxidative stress, extracellular matrix production, apoptosis, and beyond [23,48,49,50]. Glycolysis and its metabolite lactate are upregulated in a broad range of renal diseases, contributing to renal injury, inflammation, and fibrosis [20,24,51,52,53,54]. Furthermore, increasing evidence indicates that glycolysis is crucial in initiating the shift from acute kidney injury (AKI) to CKD, emphasizing the potential of inhibiting glycolysis as a therapeutic approach for this condition [48,55]. A significant body of studies has confirmed the pivotal role of glycolysis in CKD pathogenesis, revealing a positive correlation between elevated levels of glycolysis enzymes (PFKFB3, PKM2) and renal fibrosis degree, as well as Cr and BUN levels in CKD patients [56,57]. Additionally, urinary lactate levels were found to be significantly increased in CKD patients and negatively associated with glomerular filtration rate [56]. Wang et al. discovered that sustained glycolysis in RTCs leads to lactate accumulation, triggering elevated H4K12la levels which ultimately activate the NF-κb inflammatory pathway and mediate renal injury and fibrosis [56]. Cao et al. demonstrated that sustained glycolysis induces EMT in RTCs, contributing to renal fibrosis development and impairment of renal function [24]. Ding et al., using a UUO model, observed that persistent glycolysis promotes myofibroblast cell activation and exacerbates renal fibrosis [57]. However, the precise role and underlying mechanisms of glycolysis in nephrolithiasis remain largely elusive. Increased FDG uptake has been reported in the renal parenchyma adjacent to renal stones, correlating with faster stone growth in patients with higher FDG uptake [7]. This suggests a potential role of renal metabolic reprogramming in renal stone formation and associated inflammatory kidney injury. Here, our study demonstrates impaired FAO metabolism and lipid accumulation in the RTCs in human specimens with CaOx nephrolithiasis as well as in vivo experiments. In contrast, enhanced glycolysis was seen in the presence of CaOx nephrolithiasis. Similarly, in vitro experiments demonstrated an increased expression of key glycolysis enzymes and lactate production when RTCs were exposed to CaOx crystals. In conclusion, this study proves that RTCs undergo metabolic reprogramming towards glycolysis in CaOx nephrolithiasis. To elucidate the role of glycolysis in RTC injury, inflammation, and EMT, 2-DG was used. The experiments revealed that 2-DG inhibited the production of lactate, the levels of glycolysis-related enzymes, apoptosis, inflammation, and EMT in RTCs. These findings suggest glycolysis is required for kidney injury, inflammation, and EMT. At the same time, it also highlights the potential of targeted inhibition of glycolysis as an effective treatment for CaOx crystals. However, further investigations are required to elucidate the role of glycolysis and its potential mechanisms in calcium oxalate-induced renal injury and fibrosis models. Additionally, a substantial number of future clinical trials will be necessary to validate the feasibility of pharmacologically targeting glycolysis.

SMYD2, a histone methyltransferase, is involved in regulating several biological processes through the methylation of histone and non-histone substrates [30]. SMYD2 primarily targets non-histone substrates [58], including STAT3 [34], P53 [59], Erα [60], RB [61], and PTEN [62]. SMYD2 is not only upregulated in several renal diseases and mediates renal injury and fibrosis [26,29], but is also closely connected to a variety of metabolic disorders [31,32]. In this study, immunofluorescence and immunohistochemical staining of human specimens and animal models revealed an increased expression of SMYD2 and key glycolysis enzymes in kidneys with CaOx nephrolithiasis. In vivo experiments demonstrated that AZ505, a highly selective inhibitor of SMYD2, not only suppressed glycolysis but also effectively prevented stone formation, reduced renal injury and inflammation, and alleviated fibrosis. Subsequent in vitro studies demonstrated that SMYD2 siRNA inhibited the expression of glycolysis-related enzymes, attenuated apoptosis, inflammation, and EMT in vitro, and suppressed the upregulation of fibrosis-related proteins. These findings suggest that SMYD2 drives glycolysis in RTCs, thereby influencing renal injury and fibrosis in the context of CaOx nephrolithiasis. 

mTOR is a protein kinase, consisting of mTORC1 and mTORC2. It is essential for the regulation of biological processes such as cell proliferation, cell metabolism, apoptosis, and autophagy, with energy metabolism being the most important [63]. mTORC1 integrates signals from various growth factors and nutrients to promote cell growth [64]. Activation of mTORC1 promotes glycolysis by enhancing the expression and activity of PKM2, HK2, and LDHA. Hence, the mTORC1 pathway is central to glycolysis regulation [65]. PTEN is a pivotal negative regulator of mTORC1 and a potential methylation substrate for SMYD2. Several studies have shown that activation of the mTORC1 pathway by inactivating PTEN is an important factor in promoting renal injury and fibrosis. However, the mechanisms leading to reduced PTEN activity and expression remain largely unknown. Our investigations revealed that the inactivation of PTEN accompanies the activation of the mTORC1 pathway in CaOx nephrolithiasis. Inhibition of SMYD2 upregulated PTEN expression and suppressed mTORC1 pathway activation. Subsequent in vitro CO-IP analysis confirmed the interaction between SMYD2 and PTEN in COM-induced HK-2 cells, where SMYD2 inhibited PTEN expression by methylating it. Finally, we performed rescue experiments to confirm that SMYD2 regulates glycolysis via PTEN. Consequently, we confirmed that SMYD2 regulates the mTORC1 pathway through PTEN methylation, revealing a novel epigenetic regulatory mechanism in glycolysis modulation.

In summary, the results suggest that inhibiting aerobic glycolysis can protect against CaOx crystal-induced kidney injury and fibrosis. The study demonstrated the epigenetic regulatory role of SMYD2 on metabolic reprogramming in CaOx nephrolithiasis and associated renal injury. SMYD2 activates the mTORC1 signaling pathway and promotes glycolysis in RTCs by methylating PTEN. The targeted inhibition of SMYD2 and glycolysis emerges as a promising treatment for CaOx crystal-induced renal injury and fibrosis, offering potential as an effective therapy to prevent kidney stone recurrence.

## 5. Limitation

First, the SMYD2-specific knockout mouse model has not been established. Second, this work primarily focuses on the investigation of glycolytic metabolism in RTCs; however, future research efforts will include the exploration of other metabolic pathways, such as FAO, to further elucidate the regulation of energy homeostasis in RTCs.

## Data Availability

The original contributions presented in the study are included in the article/Supplementary Material, further inquiries can be directed to the corresponding author.

## Supplementary Materials

The following supporting information can be downloaded at: https://www.mdpi.com/article/10.3390/biomedicines12102279/s1, Figure S1: Research scheme on the function and mechanism of SMYD2 in regulating glycolysis induced by CaOx in RTCs; Figure S2: 2-DG rescued the decrease in cell viability caused by COM.

## Abbreviations

Abbreviations	Full Name
AKI	acute kidney injury
CaOx	calcium oxalate
CO-IP	co-immunoprecipitation
COM	calcium oxalate monohydrate
CKD	chronic kidney disease
EMT	epithelial-mesenchymal transition
LDHA	lactate dehydrogenase A
RTCs	renal tubular cells
2-DG	2-deoxy-D-glucose
HK-2	human renal tubular epithelial cells
FAO	fatty acid oxidation
HK2	hexokinase 2
PKM2	pyruvate kinase M2
IP	immunoprecipitation
ELISA	enzyme-linked immunosorbent assay
HE	hematoxylin–eosin
VK	von Kossa
