# Supplementary material for: SMYD2 Promotes Calcium Oxalate-Induced Glycolysis in Renal Tubular Epithelial Cells via PTEN Methylation
Source: Biomedicines. 2024 Oct 8;12(10):2279. doi: 10.3390/biomedicines12102279 (PMC11504487; doi:10.3390/biomedicines12102279)
Supplement: Supplementary file 1 [file biomedicines-12-02279-s001.zip › biomedicines-3193291-supplementary.pdf]

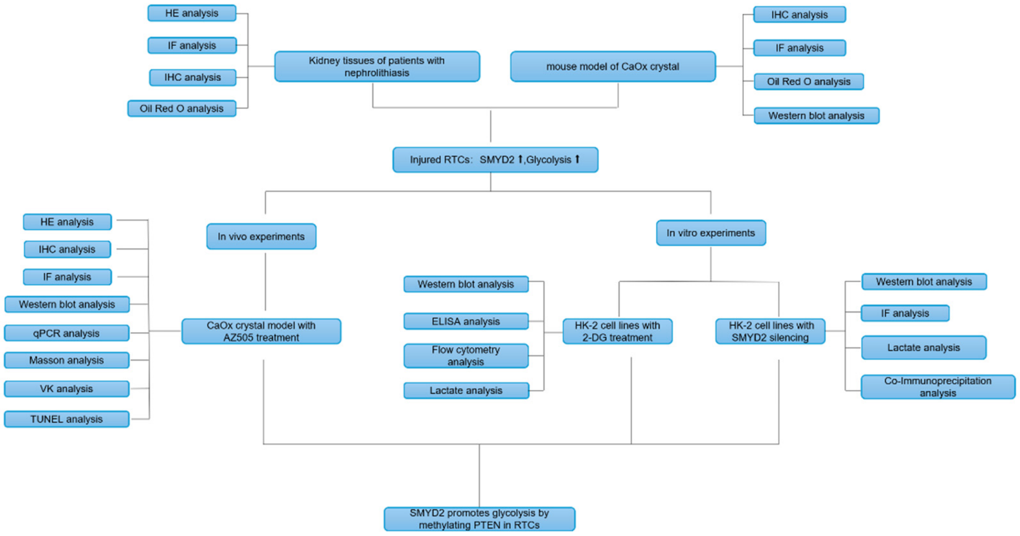

Supplementary Figure S1. Research scheme on the function and mechanism of SMYD2 in regulating glycolysis induced by CaOx in RTCs. HE:hematoxylin-eosin; IF: immunofluorescence; IHC: immunohistochemistry; VK: Von Kossa; qPCR: quantitative real-time PCR; ELISA:enzyme-linked immunosorbent assay.

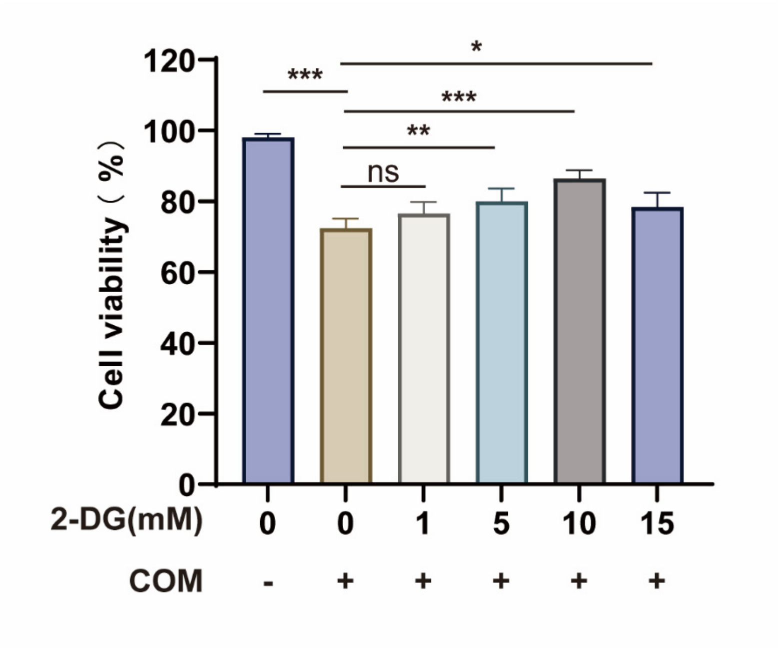

Supplementary Figure S2. 2-DG rescued the decrease in cell viability caused by COM. HK-2 cells were pretreated with 2-DG (1 mM, 5 mM, 10 mM, 15 mM) for 1 h and then treated with COM for 48 h. Cell viability was assessed by CCK8 assay. The findings demonstrate that 2-DG rescued the decrease in cell viability caused by COM, with the most obvious improvement in cell viability observed at a concentration of 10 mM.
